# Abnormalities of Penile Curvature: Chordee and Penile Torsion

**DOI:** 10.1100/tsw.2011.136

**Published:** 2011-07-28

**Authors:** Sylvia Montag, Lane S. Palmer

**Affiliations:** Division of Pediatric Urology, Cohen Children's Medical Center of New York of the North Shore-Long Island Jewish Health System, Long Island, NY, USA

**Keywords:** penis, chordee, torsion, child, surgery

## Abstract

Congenital chordee and penile torsion are commonly observed in the presence of hypospadias, but can also be seen in boys with the meatus in its orthotopic position. Varying degrees of penile curvature are observed in 4–10% of males in the absence of hypospadias. Penile torsion can be observed at birth or in older boys who were circumcised at birth. Surgical management of congenital curvature without hypospadias can present a challenge to the pediatric urologist. The most widely used surgical techniques include penile degloving and dorsal plication. This paper will review the current theories for the etiology of penile curvature, discuss the spectrum of severity of congenital chordee and penile torsion, and present varying surgical techniques for the correction of penile curvature in the absence of hypospadias.

## INTRODUCTION

Ideally, a penis should be straight; i.e., the corpora straight, the skin sufficiently lax to avert traction, and the glans with no element of torsion. Penile curvature, including chordee and penile torsion, can be found in boys with and without hypospadias. While the causes of chordee are evident in boys with hypospadias, its precise etiology, as well as that of torsion, in the absence of hypospadias, remain incompletely understood. Recent studies have furthered our understanding of the possible etiology and previously proposed explanations have been revised, which largely resulted in changes in surgical techniques. The current surgical strategies are largely successful in correcting the penis with abnormal curvature.

## EPIDEMILOGY

Penile curvature is a spectrum of disease most commonly associated with hypospadias, but is not uncommon in boys with an orthotopic meatus. The prevalence of hypospadias in the general population is approximately 1 in 300[1] and as many as one-fourth will have chordee[1]. In the U.S., the nationwide Birth Defects Monitoring Program (BDMP) reported a doubling in the rates of hypospadias since the 1970s to about 4 per 1000 in 1993[2]. Given that chordee occurs in the absence of hypospadias and that some boys are not diagnosed until later in life when the foreskin is retracted, the true incidence of chordee is substantially higher, 4–10% of male births[3,4]. Penile torsion is another curvature malformation that can be congenital and associated with hypospadias, or can be acquired after circumcision[5,6]. It results in a rotational defect of the penile shaft, most commonly in the counterclockwise direction. Isolated penile torsion has also been described[7,8], but reports are sparse, with the largest series reporting 46 cases over a 6-year period[5]. The true incidence of the deformity is unknown.

## EMBRYOLOGY OF PENILE DEVELOPMENT

The development of the penis and the urethra take place early in fetal development. The bilayered cloacal membrane (ectoderm and endoderm) becomes flanked by cloacal folds early in the 5$^{th}$ week that meet anteriorly to form the genital tubercle. The cloaca then divides into an anterior urogenital sinus and a posterior anorectal canal. The mesenchymal folds flanking the urogenital sinus become urogenital folds. The corporal bodies, connective tissue, and dermis of the penis are derived from mesodermal cells. The elongating phallus is covered with skin derived from ectoderm. The molecular mechanisms that regulate this mesenchymal differentiation likely depend on epithelial-mesenchymal interaction. Human fetal studies reveal that a ventral curvature is a normal state of penile development at the 16$^{th}$ week of gestation that resolves during the 20–25$^{th}$ week[9].

Recent studies have elucidated our understanding of the penile neurovascular anatomy. The neural supply originates under the pubic rami superior and lateral to the urethra as two well-defined bundles that travel towards the glans spreading around the corpora cavernosa to the junction with the spongiosum. This leaves the dorsal midline along the entire shaft devoid of neural tissue; this is also where the thickness/strength of the tunica albuginea is also the greatest[10].

## ETIOLOGY

### Congenital Chordee

The earliest documentation describing penile curvature dates back to Galen (130–199 AD)[11] Mettauer first defined its etiology in 1842 as “skin tethering implicating subcutaneous tissue for cause of penile curvature”[12]. Since chordee was first described in boys with hypospadias, the leading theories included: (1) abnormal development of the urethral plate, (2) presence of abnormal fibrotic mesenchymal tissue at the urethral meatus, and (3) ventral-dorsal corporal disproportion[9]. Recent studies show that ventral curvature is a normal stage of embryogenesis and, therefore, chordee without hypospadias may represent arrested penile development.

Young first described chordee in the absence of hypospadias and proposed that a congenitally short urethra was responsible[13]. In opposing this theory, Devine and Horton believed that various deficiencies of penile fascial layers contributed to penile curvature without hypospadias and proposed a classification system[14]. Kramer et al. added a fourth category of corporal disproportion in the absence of hypospadias[15]. In rare cases, a congenitally short urethra can be the etiology for ventral curvature[16,17]. Recently, a large series of congenital chordee without hypospadias was evaluated and revealed that the etiology can be evenly divided among skin tethering, fibrotic dartos and Buck’s fascia, and corporal disproportion (Table 1). The series included 87 patients with ventral (84%), dorsal (11%), and lateral (5%) curvature. Patients with thin hypoplastic distal urethra (Type I chordee based on the Devine and Horton classification system) were excluded from the study, since they were considered hypospadiac variants. A congenitally short urethra occurred in only 7% of patients[17]. Snodgrass et al. found no histological evidence of fibrous bands or dysplastic tissue in the urethral plate of boys with varying degrees of hypospadias with and without chordee; all samples demonstrated well-vascularized connective tissue comprised of smooth muscle and collagen[18], which was consistent with previous case reports[10,19].

**TABLE 1**
**Etiology for Penile Curvature without Hypospadias[17]**

| Group | Definition | No. Patients (%) | Complications (%) |
|---|---|---|---|
| 1 | Skin chordee | 28 (32) | None |
| 2 | Fibrotic fascia (dartos and Buck’s) | 29 (33) | 2 (7) |
| 3 | Corporal disproportion | 24 (28) | 2 (8) |
| 4 | Congenital short urethra | 6 (7) | 3 (50) |

Dorsal and lateral curvature of the penis occurs in cases with as well as without hypospadias[20,21]. All cases referred to children with associated hypospadias. The incidence of dorsal curvature in the absence of hypospadias is low (5%) and primarily associated with epispadias[22]. In boys, true congenital curvature has been associated with a long phallus and its correction is recommended in the case of functional impairment[22]. Dorsal chordee has been described after circumcision most likely secondary to scarring; spontaneous resolution has been reported. Repair of dorsal chordee is recommended when curvature is more than 30 degrees and/or associated with hypospadias[20].

## Penile Torsion

Congenital penile torsion is a malformation of unknown cause in which there is a three-dimensional malrotation of the corporal bodies or sometimes just the glans (Fig. 1). The abnormal penile rotation is usually counterclockwise, more common on the left side[23], and many times associated with other penile or urethral malformations, such as chordee or hypospadias. The incidence of isolated penile torsion is 1.7–27%, with torsion of more than 90 degrees reported in 0.7% of cases[24,25]. Torsion of the penis can vary in severity ranging from 30 degrees in mild cases to 180 degrees. It is hard to know how much of a functional problem this malformation causes in adults. Most children who present are asymptomatic; however, the parents usually wish to correct the cosmetic defect. In a survey of adult men evaluated at a sexual dysfunction/infertility clinic, 12% of patients had penile torsion. Of those, 80% had a mild form of the abnormal curvature (<30 degrees), 5% had torsion of more than 60 degrees, and, overall, 2% of these patients actually requested corrective cosmetic surgery. No patient complained of sexual dysfunction related to penile torsion[26].

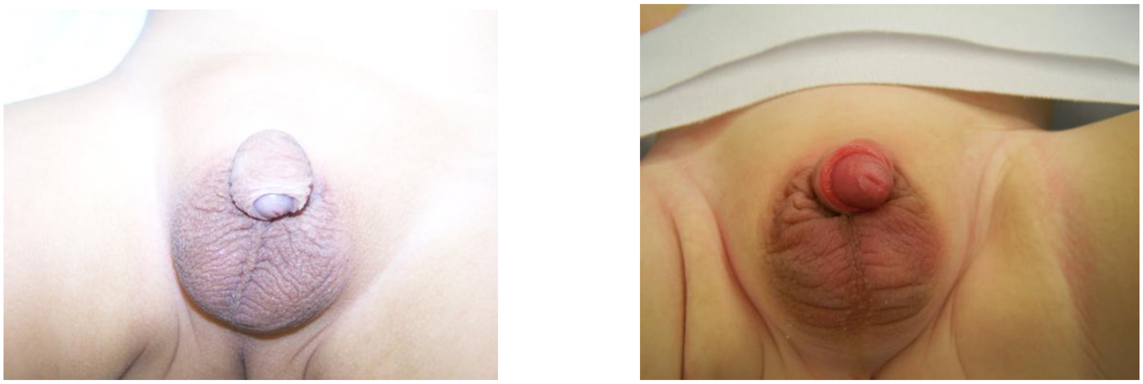

A B

**FIGURE 1.** Examples of penile torsion: (A) a milder torsion and (B) a more significant degree of torsion.

## EVALUATING BOYS WITH ABNORMAL PENILE CURVATURE

Children presenting with reported abnormal penile curvature should be assessed for the degree of curvature and/or torsion, and its direction, as well as any other genital anomalies, such as hypospadias, urethral hypoplasia, and cryptorchidism. In the clinic setting, the ability to make an assessment depends on the cooperation of the child as well as his anatomic limitations. Compression of the suprapubic fat should be performed to best expose the penis, and assess the presence or absence of chordee or torsion. It is also important to assess the prepuce, and whether the complete or incomplete prepuce might be responsible for any of the curvature. Along these lines, a tight frenulum ventrally or an epithelial skin bridge dorsally or laterally may be the cause of the curvature. If the glans penis is covered by an irreducible phimosis, then the presence of chordee may be inferred by inspecting the penis from the side of the penis and the presence of torsion may be inferred from deviation of the median raphe. The degree of chordee can be best evaluated when boys have an erection at the time of examination; unfortunately, this is not very common. Penile torsion may be assessed by using the orthotopic meatus as a guide to determine the degree of rotation. .

In the operating room, penile curvature is assessed by inducing either artificial or pharmacologic erection performed after degloving the penile shaft skin. First, it is important to reassess the curvature noted in the clinic by reducing the prepuce and identifying torsion and/or chordee, and to inspect for tethering of the glans by either the frenulum or skin bridges. The incision is made where a circumcision would be performed or use the existing line of a previous circumcision. The penile shaft must be degloved evenly at Buck's fascia to the same level proximally, while identifying and protecting the urethra with a small catheter (5F or 7F) in the urethra. A fine needle (25 Gauge) is used to inject saline into the lateral aspect of one of the corpora or through the glans with a tourniquet at the base of the penis. Artificial erection can be performed multiple times during a case with release of the tourniquet to release the erection after gleaning the desired information; however, it is important to recognize that each injection can cause a hematoma. Pharmacologic erection can be induced by intracorporal injection of a vasodilator (prostaglandin, papaverine, phentolamine) instead of saline. This technique may better evaluate chordee originating at the penile base or in cases where a large suprapubic fat pad is encountered that makes tourniquet placement difficult. The disadvantages of the pharmacologically induced erection is the lack of accurate dosing regimens in children, lack of response or prolonged erections lasting more than 6 h (priapism), additional cost, and the need for a reversal agent (phenylephrine)[27].

Similarly, the extent of penile torsion requires proper penile skin degloving. In cases where the glans is torsed due to misdirected healing of a circumcision, the glans will spring back into its normal position. In other cases, the glans will have an accentuated torsion, as the circumcision line of healing maintains the glans in a position that under-represents the true extent of torsion. Finally, the glans may remain in its position despite degloving. Once the degree and location of curvature and/or torsion has been assessed, the site for orthoplasty and the specific technique to be employed are dictated by the direction and severity of the curvature.

## SURGICAL TECHNIQUES FOR CORRECTION OF PENILE CURVATURE

### Chordee

#### *Skin Bridge and Frenular Release*

The lateral or dorsal tilting of the glans can be corrected by releasing the skin bridging or release the frenulum responsible for ventral deflection. Sometimes this is a very simple maneuver, as the skin bridge is thin and narrow, and can simply be excised and hemostasis obtained. Other times, the skin bridge is broad and thick. In these cases, it is important to secure the plane between the skin bridge and the coronal sulcus before excising the skin bridge, and then to secure hemostasis and skin closure. The surgeon must remember that the skin bridge may be only partially responsible for the curvature.

## Skin Release and Transfer

Penile skin tethering may be the sole source for mild penile curvature or low degrees of penile torsion. This is identified after proper degloving and artificial erection, if necessary. If curvature or torsion have resolved, the procedure is complete after skin closure. In some cases, any resulting ventral skin deficiency can be corrected with rotation of a pedicle preputial patch[28].

## Plication Techniques (Fig. 2)

The principle of plication is the next simplest technique for correcting curvature. Following degloving, the plication is applied opposite to the point of maximum curvature determined during the artificial erection. Heineke (1886)[29] and Mikulicz (1887)[30] described a new method of pyloroplasty closure in which a longitudinal incision was closed transversely; this was applied by Nesbit (1965), who excised diamond-shaped wedges at the point of maximum curvature[31].

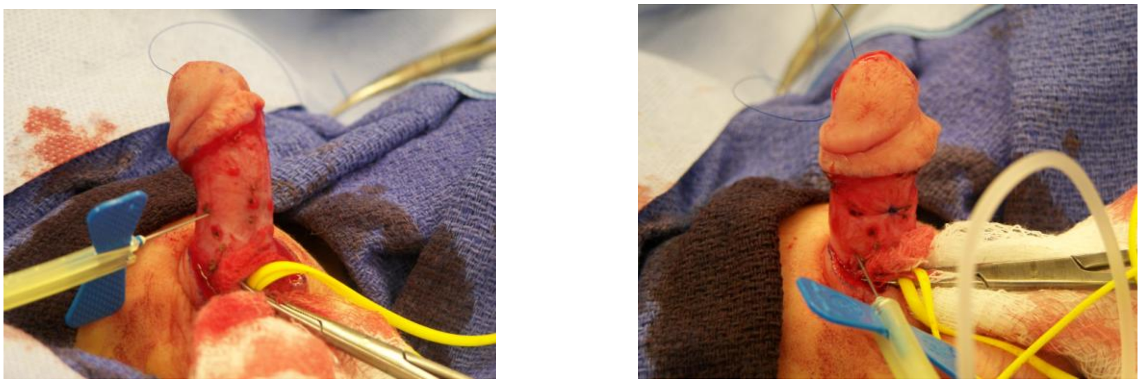

A B

**FIGURE 2.** (A) Artificial penile erection is performed by injecting saline through a fine butterfly needle (25G) directly into one of the corpora cavernosa with a tourniquet positioned at the base of the penis. (B) Plication sutures were placed at the point of maximal deflection; in this case, on the ventral surface of the penis to correct the dorsal chordee. The repeat artificial erection shows adequate correction of the curvature.

It has become clearer from recent anatomical studies that any dissection of the neurovascular bundle may damage nerve fibers that fan out from the 11 and 1 o'clock positions towards the ventral surface[10,32]. The only area truly devoid of nerve fibers is the 12 o'clock position, which also appears to be the area of greatest tunica albuginea thickness and strength[10,33]. Dorsal midline plication with one or multiple parallel sutures was described by Baskin et al.[10] to ensure maximum preservation of nerves and has since been applied by others with good short-term results[34,35]. Potential disadvantages with this technique include limited applicability to mild-moderate penile curvature and poor efficacy when used in older boys, as the midline plication sutures will probably not hold up to rigid erections[36].

## *Dermal and Other Grafts (Fig. 3)*

In 1975, Devine and Horton described their experience adding dermal tissue to the tunica albuginea to correct chordee associated with hypospadias and epispadias[37]. Since then, several other authors have reported their similar success[38,39,40]. When significant curvature persists after penile degloving, the surgeon has to decide whether plication can correct the chordee without significant shortening of the penis or whether a dermal graft may be used. The urethra can be separated from the corporal bodies, or transected if there is a hypospadias. An artificial erection is created and the tunica albuginea is incised at the point of maximum curvature, including the septum between the corporal bodies. Care must be taken not to incise cavernosal tissue and nerve tissue must be preserved on the lateral aspect of the phallus[42]. Dermal grafts are prepared by excision of an elliptical segment of non–hair bearing skin from the inguinal region. Once the epidermis is removed by shaving it from the dermis using sharp scalpels, the dermis is then defatted. The remaining dermis is trimmed to the size of the defect and attached to the edges of the tunica albuginea with absorbable suture. Repeat artificial erection should be obtained to ensure complete orthoplasty and to confirm the absence of leakage[39,41]. The use of dermal grafts has been shown to yield superior cosmesis and can prevent penile shortening seen with extensive plication in patients with severe penile curvature[39]. Badawy and Morsi[41] reported their 10-year follow-up data for 16 patients with penile dermal grafts showing that erectile function was well preserved in 88% of patients. Two patients had mild residual curvature and one of them needed phosphodiesterase inhibitors to achieve rigid erections.

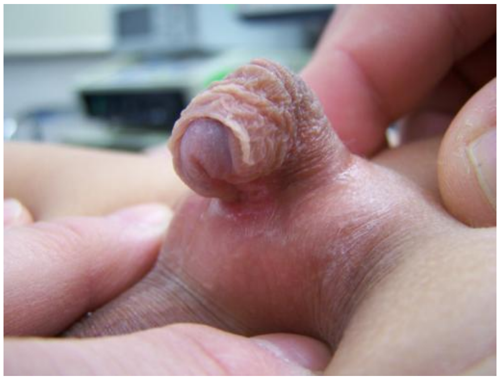

**A**

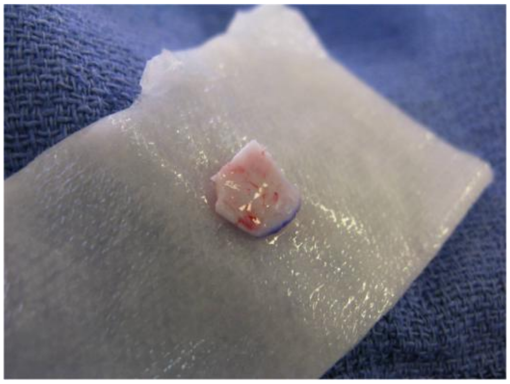

**B**

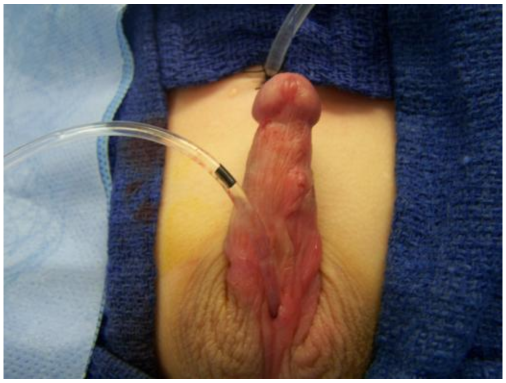

**C**

**FIGURE 3**. (A) This child had a severe ventral chordee and proximal hypospadias. (B) His chordee correction was performed by dividing the urethral plate and incising the tunica albuginea, and then placing the dermal graft. Byars skin flaps were developed from the dorsal prepuce and rotated to the ventral surface. (C) The result 6 months later is a straight penis ready for urethroplasty.

Other materials, including tunica vaginalis[40,42], dura, and pericardium[42], have also been used, but with inferior results and without long-term follow-up. Early experience with small intestine submucosa indicates that this material is safe, but its long-term durability remains unknown[43,44].

### Corporal Rotation and Penile Disassembly

For more complex curvature, corporal rotation, as described by Koff and Eakins[45], or penile disassembly, as popularized by Perovic and Djordjevic[46], offer satisfactory surgical results. The principle of corporal rotation involves separation of the corpora cavernosa from the urethral plate and distal corpus spongiosum, starting at the glans and dissecting distally. The midline septum is incised longitudinally. The neurovascular bundles are also elevated off the corporal bodies to avoid possible crush injury. The technique was further developed by adding a series of transverse dorsal plication sutures. No incision is made into the corporal bodies preventing penile shortening[47,48]. Reported series consist only of a small number of patients and long-term follow-up data are lacking.

An extension of the corporal rotation is the penile disassembly technique, as used in boys with complete primary epispadias. The penis was straightened in 68% of cases and additional minor corporoplasty was needed in one-third of patients. A major disadvantage is the extensive dissection needed to separate the dorsal nerves off the corpora, although theoretically this can be achieved with potential damage to only small side branches[32].

## Penile Torsion

In mild forms of penile torsion (<90 degrees), the glans is directed away from the midline, but the orientation of the corporal bodies at the base of the penis is usually normal. The defect is often correctable by penile degloving and realignment of the median raphe. Bar-Yosef et al.[5] reported satisfactory results in 95% of patients with isolated penile torsion with rotation <90 degrees and residual torsion of <30 degrees in 5% of patients using a simple technique of penile degloving and realignment. In children with higher degrees of torsion (>90 degrees) or torsion associated with hypospadias, the use of a dorsal rotational dartos flap may help to correct the defect. The technique first described by Fisher and Park[8] showed that at short-term follow-up, cosmetic outcomes were satisfactory in all eight patients. No complications or evidence of residual torsion were reported. In this technique, a dorsal dartos flap is rotated around the right side of the penile shaft to correct for counterclockwise (rotation to the left) torsion. The technique was successfully replicated by Bauer and Kogan[49] and none of the 25 patients needed further repair. Torsion was completely corrected in 16 patients, with the reminder of patients having an insignificant (<30 degrees) amount of residual torsion. However, long-term results for this approach are still lacking. More extensive repair may be needed for children with associated hypospadias or chordee. Bhat et al.[50] described a series of 27 cases with congenital penile torsion ranging from 45 to 180 degrees (mean 69). Only 3.7% of cases were corrected with simple skin rearrangement. The authors describe the use of extended mobilization of the urethra or even the urethral plate to correct the degree of torsion and their overall success rate was 87.5%. In this series, more extensive repair was most likely required secondary to the presence of chordee as well as hypospadias. Isolated penile torsion should be approached conservatively and if cosmetic correction is requested, the least-invasive approach should be used to correct the defect.

## SUMMARY

Penile curvature can present a challenging problem to the pediatric urologist. Better understanding of penile neurovascular anatomy has led to improvement of surgical techniques and outcomes for treatment of penile curvature. Several surgical techniques have evolved; however, none are without complications and long-term follow-up studies are lacking. The majority of congenital penile curvature without hypospadias can be corrected with simple degloving or plication techniques. Residual curvature can be addressed with additional plication or more extensive surgical correction, such as dermal graft placement and, in rare cases, complete penile disassembly. The most important aspects of any technique are preservation of the neurovascular structures and urethral plate whenever possible. Correction of curvature should be done preferably during the 1st year of life with a stepwise, minimally invasive approach. Long-term studies to assess efficacy and complications are needed in order to verify current surgical techniques.
